# Supplementary material for: Unveiling Another Dimension: Advanced Visualization of Cancer Invasion and Metastasis via Micro-CT Imaging
Source: Cancers (Basel). 2025 Mar 28;17(7):1139. doi: 10.3390/cancers17071139 (PMC11988112; doi:10.3390/cancers17071139)
Supplement: Supplementary file 1 [file cancers-17-01139-s001.zip › cancers-3307988-supplementary.pdf]

SUPPLEMENTARY S1. Design of the experiment.

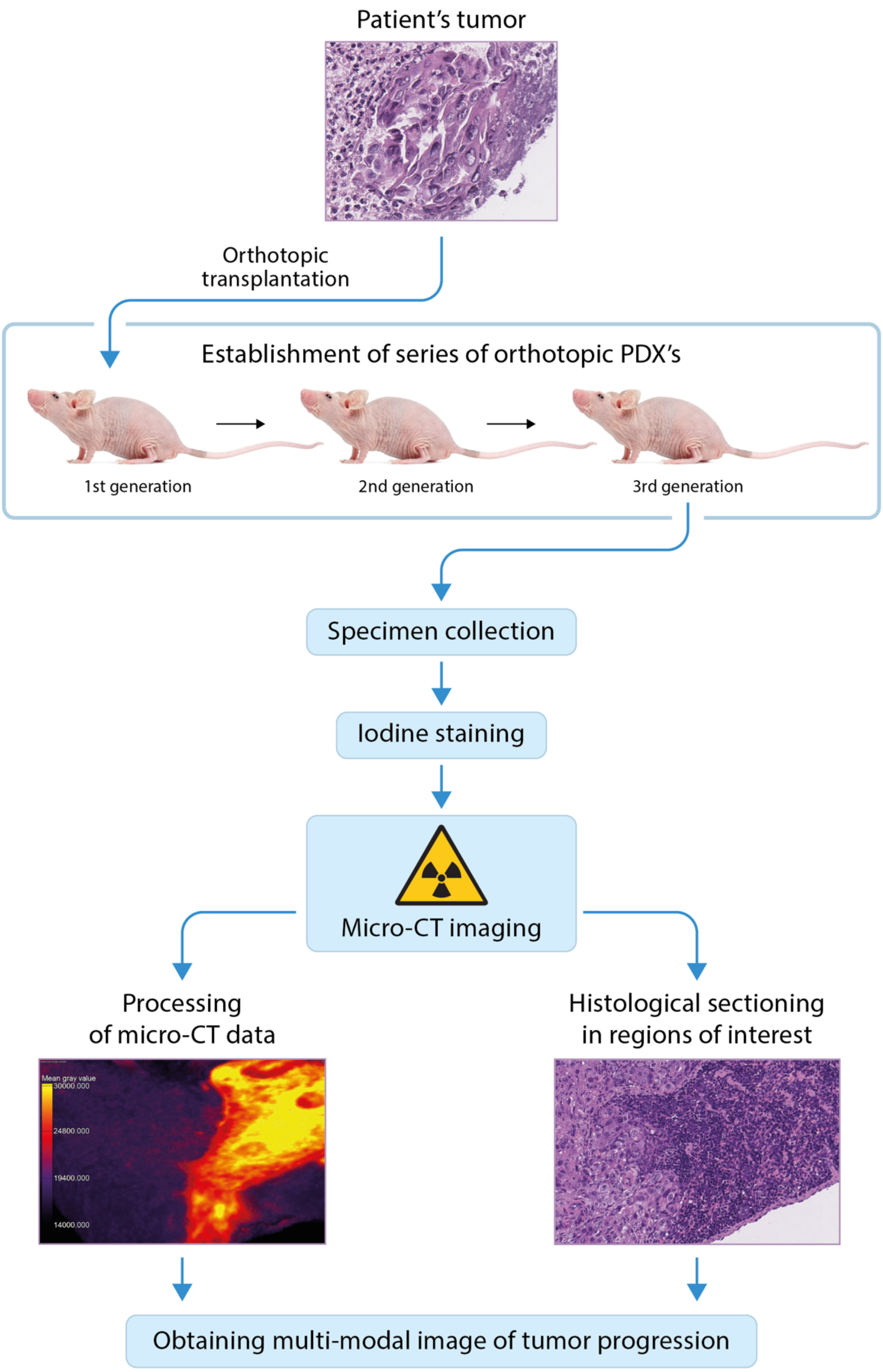

Supplementary Figure S1. Design of the experiment.

SUPPLEMENTARY S2. Acquisition parameters for different parts of the samples.

Figure	Magnification objective	Filter	Voltage, kV	Power, W	Exposure, s	Voxel size, µm	Number of projections
Figure 1 B; Figure 2 C, E; Figure 3 A; Figure 4 C; Figure 5; Figure 6 A; Figure 7 E, F; Figure 8 A, B; Figure 9 B; Supplementary Figure S4, Supplementary Figure S6.	0.4x	LE 2	50	4	3.5	10	2001
Supplementary Figure S5	0.4x	LE 2	60	5	5	5	1601
Figure 2 F; Figure 6 C;	0.4x	LE 2	60	4	1	9.5	1601
Figure 8 D; Figure 10 C; Figure 11 A, B, C, E.	4x	LE 2	50	4	2	4	4501
Figure 3 C; Figure 4 A; Figure 10 A.	0.4x	LE 2	60	5	6	5	2001
Figure 7 A, D; Figure 8 C; Supplementary Figure S2	0.4x	LE 2	40	3	5	30	3201
Figure 1 A;	0.4x	LE 2	60	5	5	5	2001
Figure 4 E; Supplementary Figure S2	0.4x	LE 2	60	5	1	19.3	1601
Supplementary Figure S3	4x	LE 2	50	4	2.5	4	3201

Supplementary Table S1. X-ray projection acquisition parameters for the objects presented in the figures.

SUPPLEMENTARY S3. Examples of tumor spread and site-specific invasion patterns.

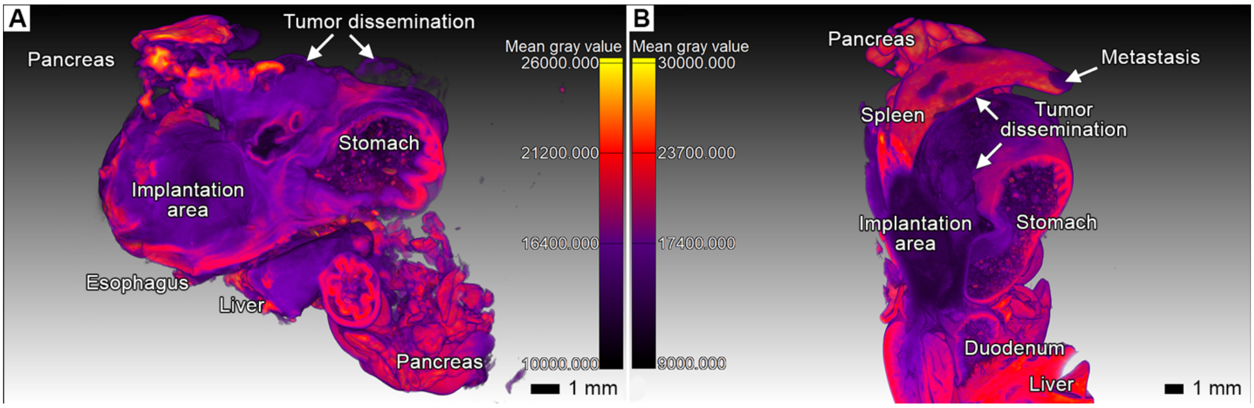

Supplementary Figure S2. 3D overview scans of the abdominal organs in both animals demonstrating tumor dissemination: **A**, specimen 1; **B**, specimen 2.

For overview scans, we used a relatively low resolution of 19.3 µm/voxel and 30 µm/voxel, respectively. While this resolution does not provide sufficient detail to precisely delineate the interface between tumor and healthy tissue, it allows for a clear visualization of the spatial distribution of tumor progression. To differentiate tumor-affected regions, we applied a color-coding method based on gray value distribution (Gray Value Analysis). This enabled us to highlight the primary tumor lesion, peritumoral area, invasion sites, and metastatic lesions, as reflected by variations in gray-level intensity. Tumor tissues exhibit a markedly lower X-ray density compared to healthy tissues, appearing as darker regions in the 3D reconstructions. This difference allows for the identification of potential regions of interest for high-resolution micro-CT imaging or histopathological validation. In our analysis, regions with mean gray values below 16,400 (specimen 1) and 17,400 (specimen 2) were predominantly associated with tumor tissue. Scale bar: 1 mm.

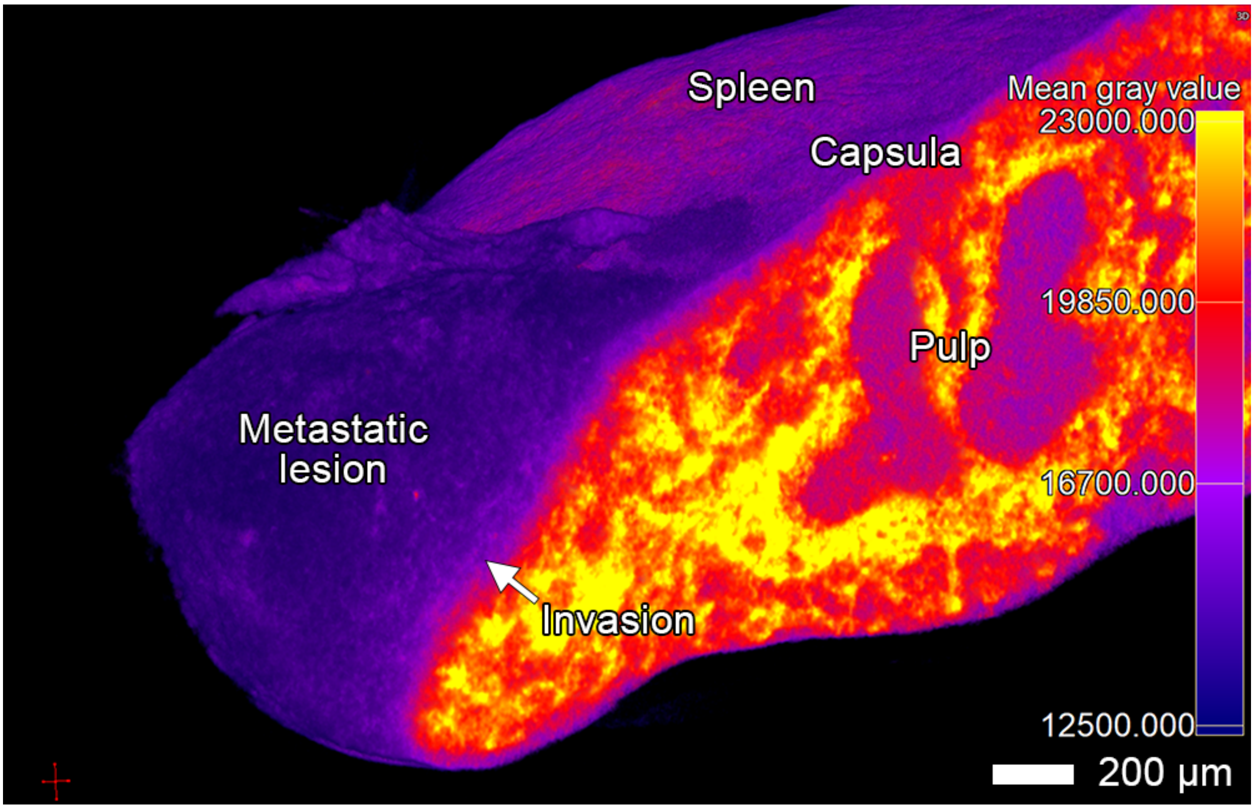

Supplementary Figure S3. Metastatic lesion in the dorsal extremity of the spleen from specimen 2. Scale bar, 200 µm.

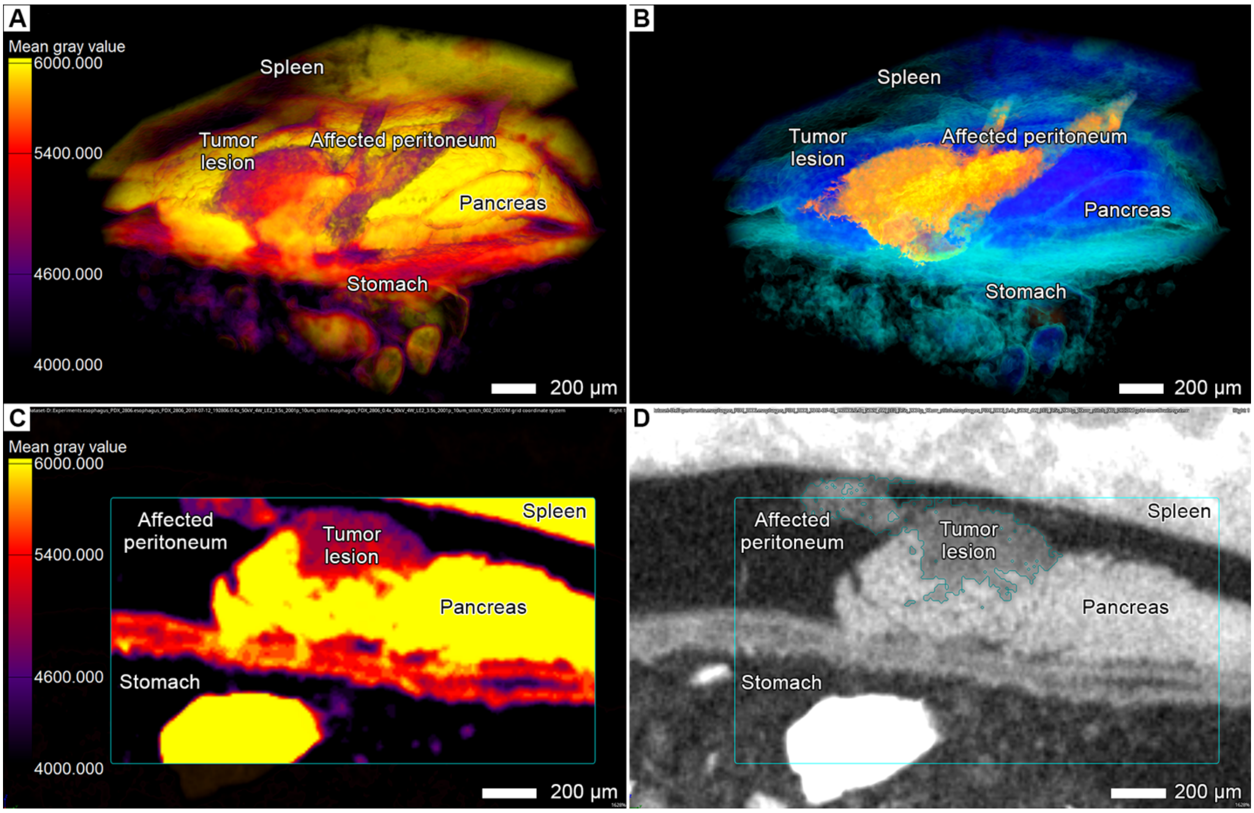

Supplementary Figure S4. Transperitoneal invasion into the pancreas, shown using two different imaging approaches. **A.** Three-dimensional visualization with Gray Value Analysis applied to the entire region of interest (ROI). **B.** Manual segmentation of the tumor using the "Region Growing" tool, based on gray value thresholds. Tumor tissue is color-coded yellow and orange, while surrounding healthy tissue appears turquoise and blue. **C.** Two-dimensional visualization of the same dataset, with the ROI highlighted as a rectangle and color-coded for clarity. **D.** Tumor and healthy tissue were differentiated using the "Region Growing" tool, leveraging the tumor's lower X-ray density, which enables segmentation based on gray value differences. Scale bar: 200 µm.

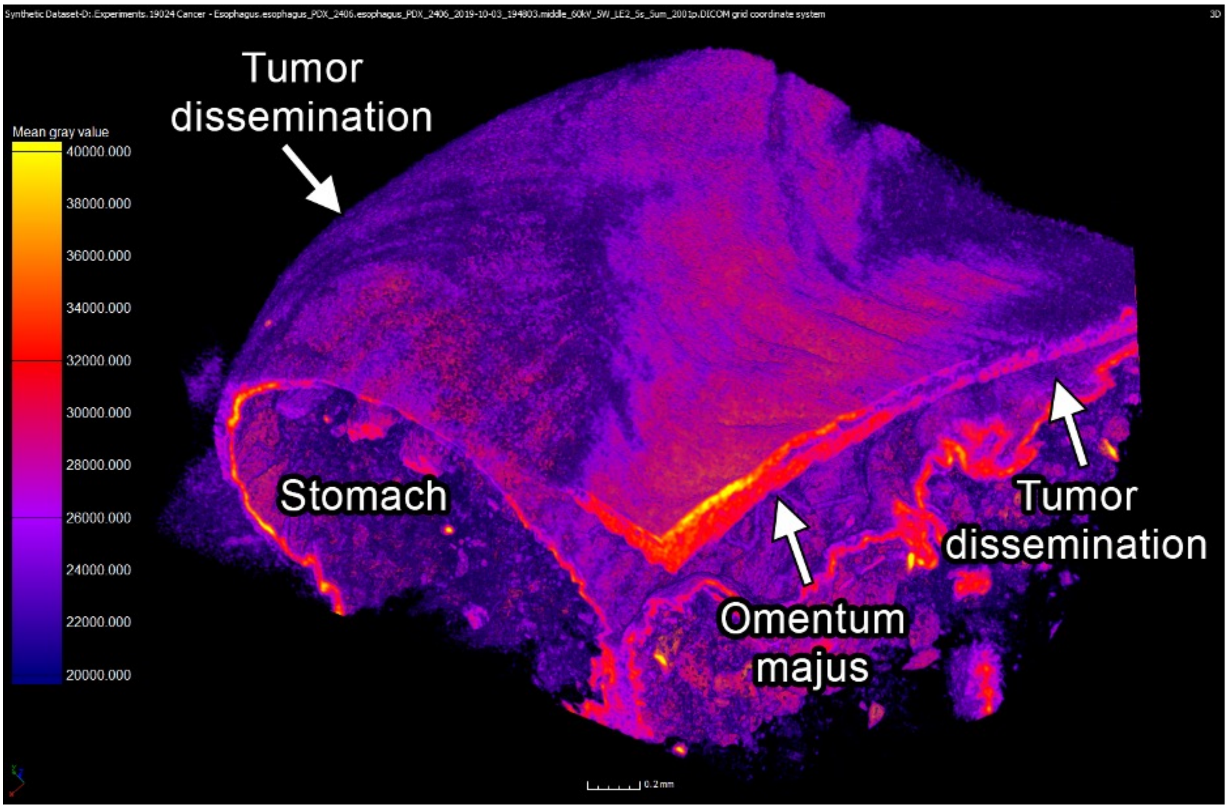

Supplementary Figure S5. Tumor cell dissemination through the omentum majus, a large fold of the visceral peritoneum extending from the stomach. Tumor cells appear as low-intensity zones, reflecting their reduced X-ray density relative to surrounding tissues. Scale bar: 200 µm.

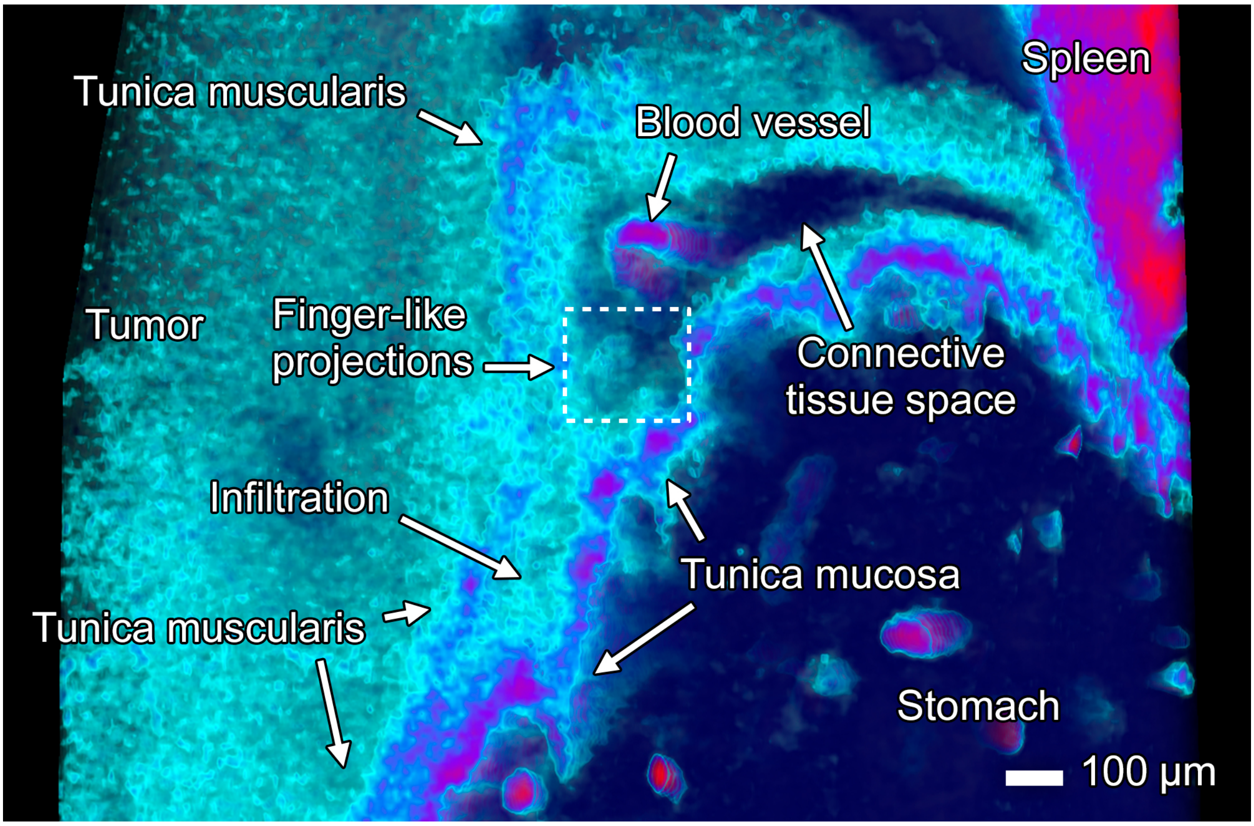

Supplementary Figure S6. Detailed three-dimensional reconstruction of the stomach invasion site. The tumor infiltrated the gastric wall, causing separation of the tunica muscularis from the tunica mucosa and forming foci of tumor infiltrate between the layers. As invasion progressed, the tumor developed finger-like projections that penetrated the tunica muscularis and extended into the connective tissue of the submucosa. This region corresponds to the same area shown in Figure 2C, 2D, and Supplementary figure 7A. Scale bar: 100 µm.

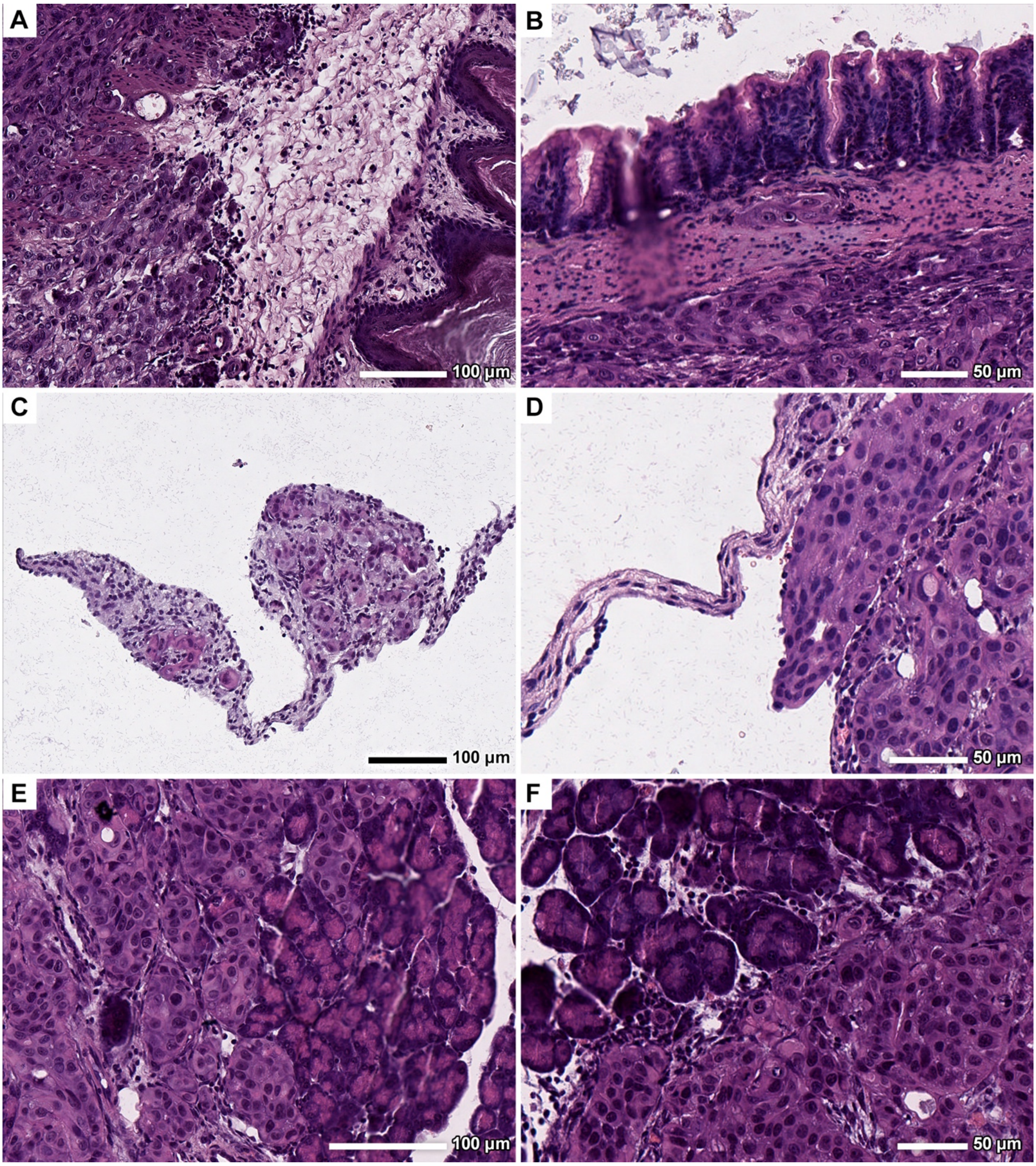

Supplementary Figure S7. Various modes of invasion across peritoneal cavity.

Depending on the tumor location, we identified the following invasion patterns:

A. As the tumor mass infiltrated the loose connective tissue of the stomach submucosa, a notable shift in the invasion pattern was observed. Initially, the tumor displayed a trabecular growth pattern, but as it progressed, cell cohesion was lost due to the disruption of intercellular contacts. This led to the formation of finger-like projections, which subsequently fragmented into individual cells and small clusters.

This histological area is notable for the clear visualization of tumor cell migration, occurring both as cohesive clusters and individual units, through the intricate network of connective tissue. Notably, both histological images and micro-CT images (Figure 2C, 2D, and Supplementary Figure 6) distinctly show how the tumor advanced into the submucosa via finger-like projections that later separated within the connective tissue. Scale bar: 100 μm.

B. Similar tumor clusters were observed in the submucosal layers of the stomach and small intestine, though they were larger (~4-5 cells, without discrete cells) due to the presence of dense fibrous connective tissue. Many of these clusters were later identified in micro-CT images, showing a clear structural connection to the invasion front (e.g., Figure 11). Scale bar: 100 μm.

C. Carcinomatosis (peritoneal metastases) exhibited similar budding patterns but was embedded within fibrotic stroma with lymphoplasmacytic and histiocytic inflammatory infiltration (desmoplastic reaction/tumor microenvironment). Scale bar: 100 μm.

D. In other areas of carcinomatosis, the tumor exhibited a predominantly solid-trabecular invasion pattern. Scale bar: 50 μm.

E, F. In pancreatic invasion, tumor cells followed a nesting pattern, colonizing acini and integrating into their fibrous framework. Scale bar: 100 μm (**E**), 50 μm (**F**).

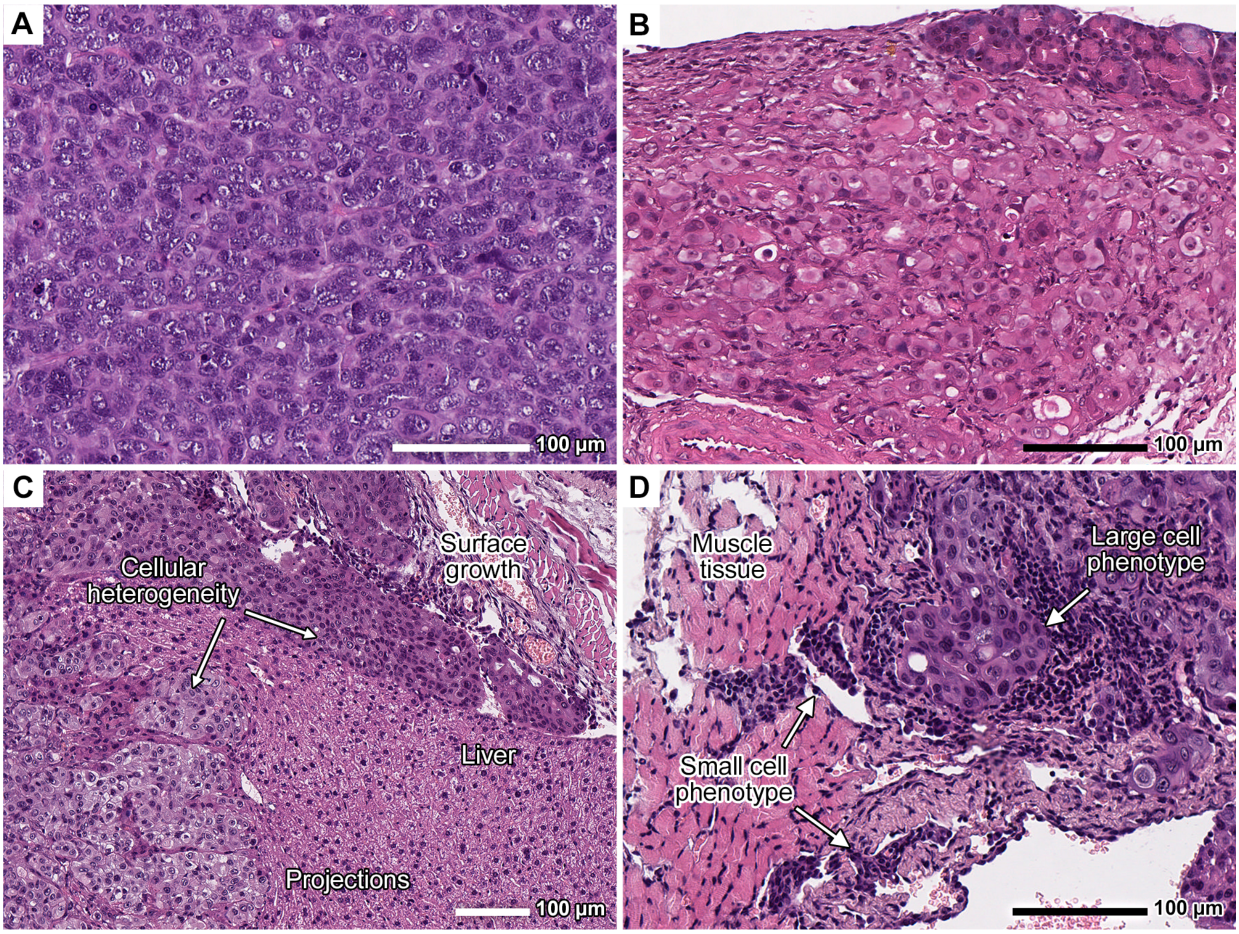

Supplementary Figure S8. The tumor exhibit cellular heterogeneity in different areas of animals.

A. The most malignant histological component consists of solid, non-keratinizing sheets of large polygonal cells with scant cytoplasm, hyperchromatic pleomorphic nuclei with nucleoli, and frequent pathological mitoses, indicative of an undifferentiated/anaplastic phenotype. Scale bar: 100 µm.

B. The second histological component is composed of dense, non-keratinizing sheets of large polygonal cells with abundant eosinophilic cytoplasm (lacking significant keratinization), large hyperchromatic moderately pleomorphic nuclei, consistent with a poorly differentiated squamous phenotype. Scale bar: 100 µm.

C. Two distinct tumor invasion patterns with different morphological characteristics, potentially reflecting adaptive phenotypic plasticity:

1. Infiltrative growth into the liver parenchyma (finger-like projection) – Tumor composed of non-keratinizing solid-trabecular structures with large epithelial cells, moderately

abundant amphiphilic cytoplasm, large hyperchromatic nuclei with nucleoli, and no keratinization (lower left corner).

2. Surface growth along the liver capsule – Tumor predominantly solid in structure, composed of smaller epithelial cells, sparsely eosinophilic cytoplasm, and medium-sized hyperchromatic nuclei, without keratinization (upper right corner).

The presence of morphologically distinct phenotypes within the same anatomical area may indicate adaptive plasticity in response to microenvironmental conditions, enabling tumor cells to optimize their mode of progression depending on the surrounding tissue architecture. Scale bar: 100 µm.

D. Two separate tumor subpopulations displaying morphological and phenotypic diversity, further supporting adaptive phenotypic plasticity:

Right side: Tumor consists of non-keratinizing sheets of large polygonal cells with moderately abundant eosinophilic cytoplasm, hyperchromatic pleomorphic nuclei with nucleoli, and a dense immune (lymphocyte) infiltrate, indicative of a large-cell phenotype.

Left side: Tumor consists of dense diffuse sheets of small cells with scant cytoplasm, hyperchromatic nuclei, and absent nucleoli, demonstrating infiltrative growth into the muscle layer and the presence of a tumor embolus, characteristic of a small-cell phenotype. Scale bar: 100 µm.

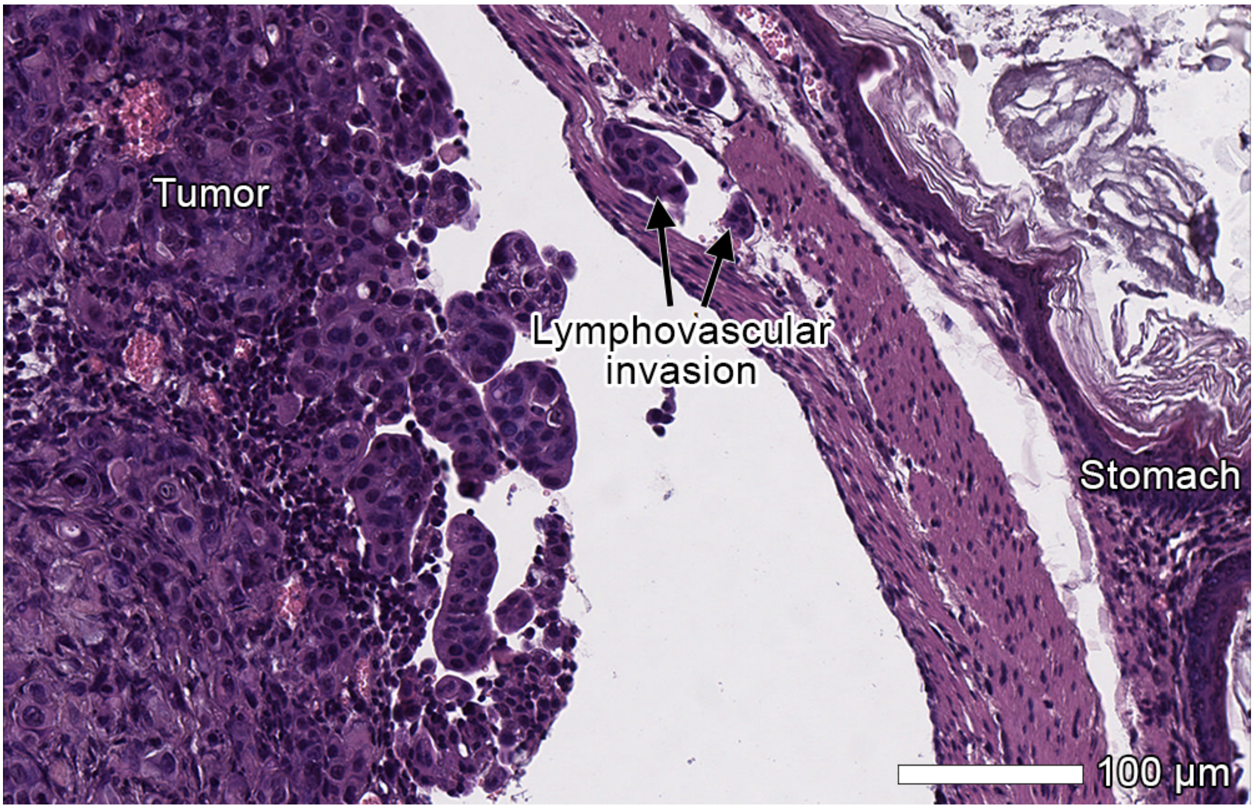

Supplementary Figure S9. Lymphovascular invasion in the nonglandular part of the stomach wall. The light micrograph reveals clusters of tumor cells within the lumen of lymphatic vessels, indicating active lymphatic dissemination. This suggests that the tumor may utilize the lymphovascular system as a route for metastatic spread. Scale bar: 100 µm.

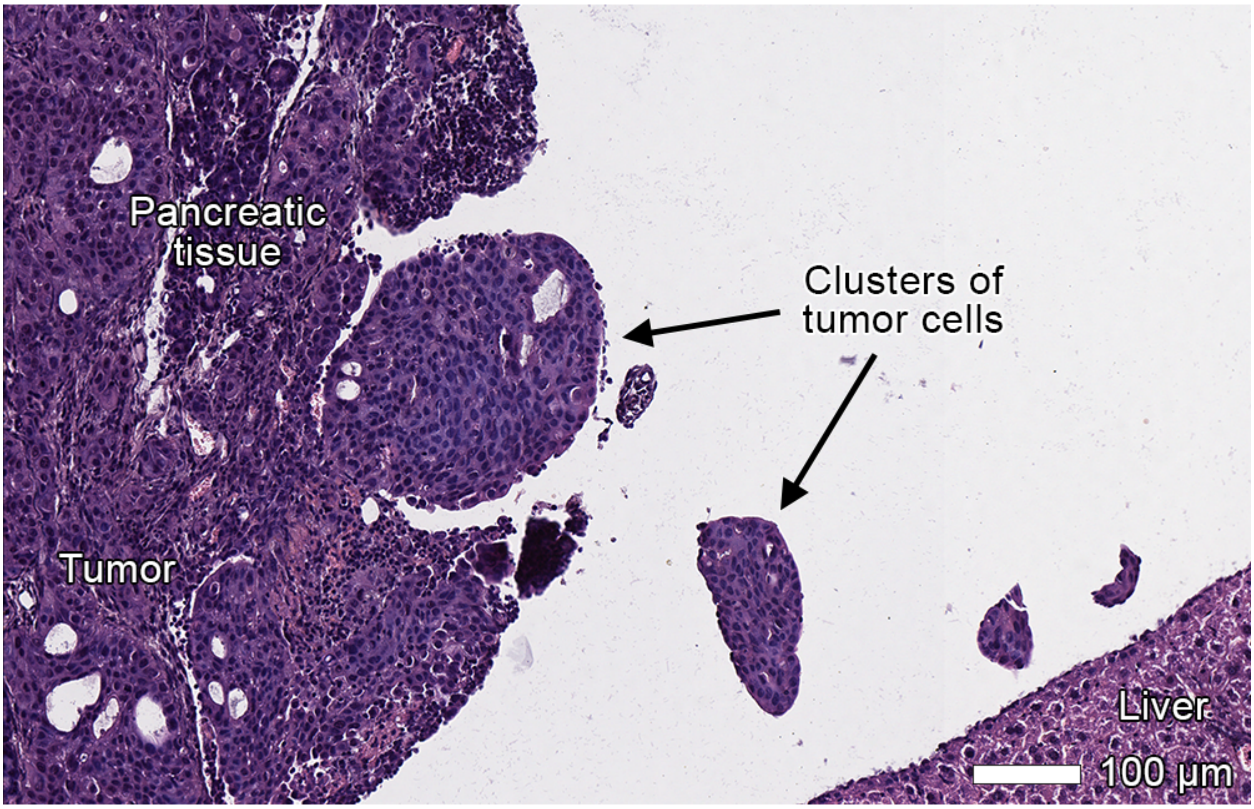

Supplementary Figure S10. Tumor clusters protruding into the peritoneal cavity. While some of these structures may be artifacts, the presence of smooth edges without signs of detachment in most clusters suggests that they are naturally occurring and formed *in vivo*. We propose that these clusters represent a potential source of free-floating peritoneal cancer cells and multicellular aggregates that contribute to peritoneal metastasis formation. Scale bar: 100 µm.
